# Quantitative assessment of distal radioulnar joint stability with pressure-monitor ultrasonography

**DOI:** 10.1186/s13018-019-1237-3

**Published:** 2019-06-27

**Authors:** Yuichi Yoshii, Hiroshi Yuine, Wen-lin Tung, Tomoo Ishii

**Affiliations:** 1 Department of Orthopaedic Surgery, Tokyo Medical University Ibaraki Medical Center, 3-20-1 Chuo Ami, Inashiki, Ibaraki, 300-0395 Japan; 2 Department of Rehabilitation, Tokyo Medical University Ibaraki Medical Center, Ami, Ibaraki, 300-0395 Japan; 3 Department of Occupational Therapy, Ibaraki Prefectural University of Health Sciences, Ami, Ibaraki, 300-0394 Japan

**Keywords:** Distal radioulnar joint, Ultrasound, Pressure, Stability

## Abstract

**Background:**

Diagnosing distal radioulnar joint (DRUJ) instability remains a challenge as it relies on physical examination. To quantitatively assess DRUJ stability, a pressure-monitor ultrasound system was developed. The objective of this study was to evaluate the force-displacement relationship of DRUJ in normal subjects.

**Methods:**

Nine wrists of 9 asymptomatic volunteers were evaluated. The pressure-monitor ultrasound system was developed to apply pressure to the tissue with a pre-determined cycle and displacement of the transducer. Each subject was imaged sitting with the elbow flexed and forearm pronated. The dorsal surface of the distal radius and the center of the ulnar head were displayed at DRUJ level. The pressure toward palmar direction was applied to the distal ulna with different levels of transducer displacements, i.e., 1 mm, 2 mm, and 3 mm. The distance between the dorsal surface of the ulnar head and the dorsal surface of the distal radius was measured. The first measurement was performed at the initial position, and the second measurement was performed when the transducer pressed down the ulna to the degree that the ulnar head had shifted to the most palmar position. At the same time, the pressure to the transducer was measured. The changes of radioulnar distance (=the measurement at the most palmar position—the measurement at the initial position) and pressure, and pressure/distance ratio were compared among the different transducer displacements.

**Results:**

The pressure was significantly increased as the transducer displacement became larger (*P* < 0.01). The changes of radioulnar distance were smaller in the 1 mm displacement condition compared to the 2 and 3 mm displacement conditions (*P* < 0.05). The pressure/distance ratio was larger in the 1 mm displacement condition compared to the 2 and 3 mm displacement conditions (*P* < 0.05).

**Conclusions:**

A method to assess DRUJ stability by measuring changes in radioulnar distance and force application was developed. It was found that the application of 2 mm displacement and 200 g force was the critical stress for the capsuloligamentous structures to start stabilizing DRUJ. This methodology and the indices may be clinically useful to investigate the mechanical properties of patients with DRUJ instability.

## Background

Diagnosing distal radioulnar joint (DRUJ) instability remains a challenge because it relies on a physical examination. Since there is an increasing trend to treat DRUJ instability surgically, it is important to evaluate DRUJ instability accurately. It is known that DRUJ stability is found in the combination of bony anatomy, DRUJ capsule, musculotendinous structures, and ligamentous structures [1–10]. Because of these complex wrist-stabilizing structures, it is difficult to understand the pathological conditions for each structure.

Generally, DRUJ instability is assessed by several manual stress tests, such as the ballottement test, ulnocarpal stress test, and piano-key test. A previous biomechanical study using cadaver wrists demonstrated that the DRUJ ballottement test was the most reliable for evaluating the instability compared with other manual stress tests [11–13]. This clinical test evaluates the translation of the distal ulna relative to the distal radius in neutral forearm rotation by applying a dorsovolar shear force. An increased translation in comparison to the healthy side wrist may be caused by pathological conditions such as TFCC injury. Although this assessment can detect gross instability, this is a qualitative and subjective evaluation. Therefore, there are clinical needs to develop a method for the quantitative assessment of DRUJ instability.

There have been several studies for the evaluations of physiological translations for the DRUJ [14–22]. Some studies evaluated clinical DRUJ instability with computed tomography (CT) [9, 14]. However, these methods cannot evaluate real situations with a dynamic force-displacement relationship. If it were possible to clarify the force-displacement relationship of DRUJ, it would be helpful to understand how much load the joint can withstand, in addition to discriminating pathological conditions quantitatively. It is important to develop a clinical method to evaluate the force-displacement relationship of DRUJ. To quantitatively assess DRUJ stability, a pressure-monitor ultrasound system was developed. In this study, we aimed to evaluate the force-displacement relationship of DRUJ in healthy volunteers and to characterize normal DRUJ stability.

## Methods

The protocol for this study was reviewed and approved by our Institutional Review Board (No17-26). Nine wrists of nine asymptomatic volunteers (nine males; age range 23–35, mean age 28.0 years) were evaluated by a pressure-monitor ultrasound system. Volunteers were excluded if they had a history of previous wrist trauma and wrist pain. Written consents were obtained from all study participants. All subjects were confirmed to have no instability in DRUJ with the ballottement test.

### Pressure-monitor ultrasound system

**Fig. 1:**
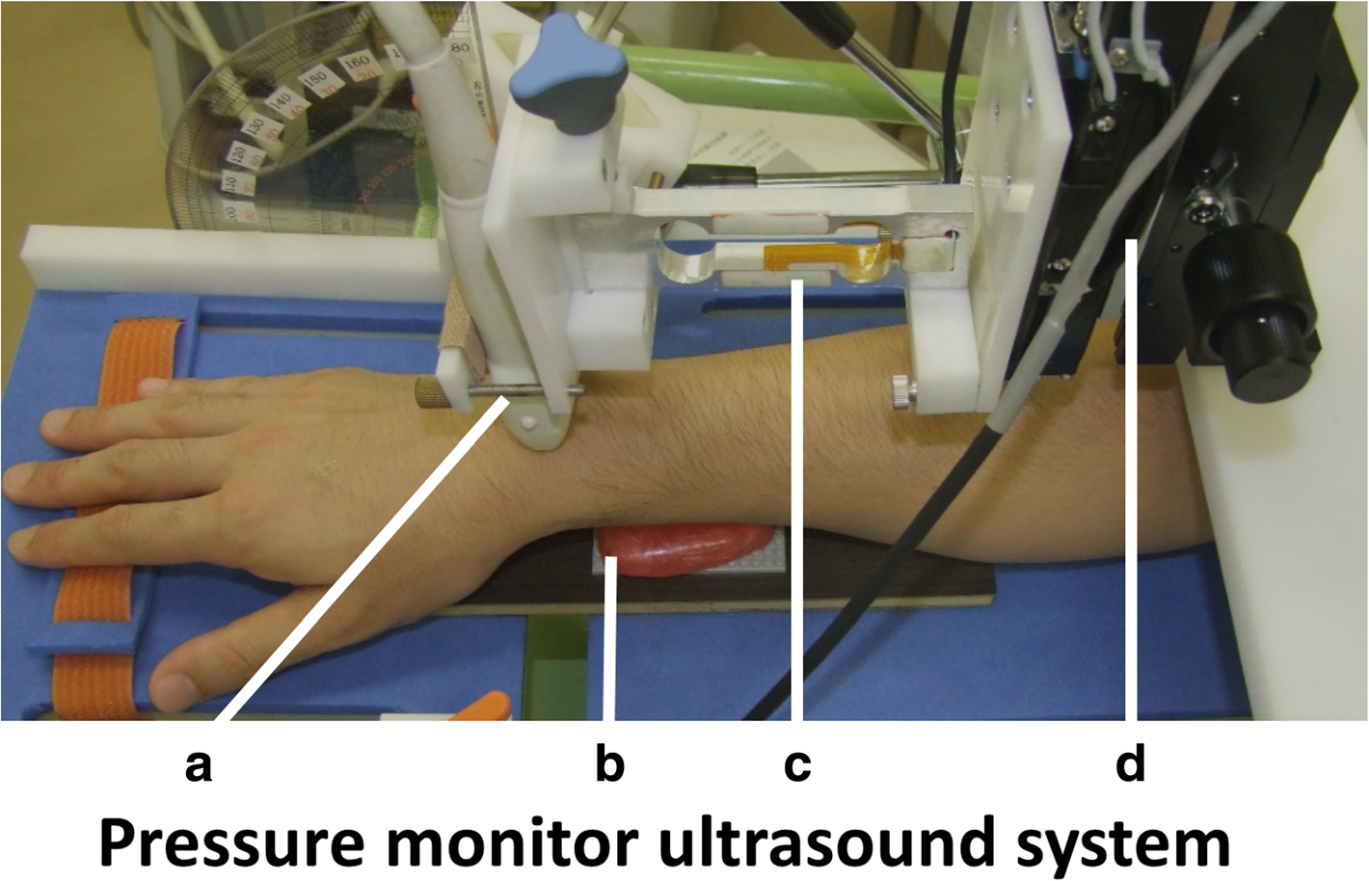
Pressure-monitor ultrasound system and the setup for the measurements. The pictures show the forearm position. The pressure-monitor ultrasound system was developed to apply pressure to the tissue with a pre-determined cycle and transducer displacement. **a** Transducer, **b** block, **c** pressure sensor, and **d** motor

The pressure-monitor ultrasound system was developed to apply pressure to the tissue with a pre-determined cycle and transducer displacement. The apparatus has two parts: a forearm table with a motor for the compression-release cycle, and a programmed controller (Fig. 1). The table was designed so that the transducer could be placed at the level of the subject’s wrist and the placement adjuster could adjust the height of the transducer. The programmed controller could adjust the displacement and cycles in the range of 0.1–3.0 mm and 0.46–4.80 Hz (Gyouden Co., Tsukuba, Japan). For pressure measurement, a pressure sensor and a force processor (F381A, Unipulse, Co.) were attached to the system. This dynamic force processor was integrated with a strain gauge sensor that could measure waveforms of physical quantities, including pressure, load, and torque, and could take measurements at a frequency of 4000 times/s, which were saved as digital data on an SD card.

### Evaluations

Each subject was tested sitting with the elbow flexed and the forearm pronated. The forearm of the examinee was secured to the table. The hand was positioned on an adjustable block such that the forearm was in a horizontal position. To stabilize the distal end of the radius, the blocks were placed below the distal radius and the pisiform bone. An ultrasound scanner (Hivision Avius; Hitachi Aloka Medical, Ltd., Tokyo, Japan) equipped with a linear array transducer was set to a depth of 20 mm. At the distal radioulnar joint level, the transducer was maintained perpendicular to the longitudinal axis of the ulna (Fig. 1). The dorsal surface of the distal radius and the center of the ulnar head were displayed on a monitor. To determine the same measurement level in each volunteer, the highest aspect of the ulnar head was taken. The DRUJ motion during cyclic loading toward the palmar direction at the distal ulna was recorded. The compression-release cycles were applied with the pressure-monitor ultrasound system. The cycle was set to 1.5 Hz. The pressure in the palmar direction was applied with different levels of transducer displacements, i.e., 1 mm, 2 mm, and 3 mm.

**Fig. 2:**
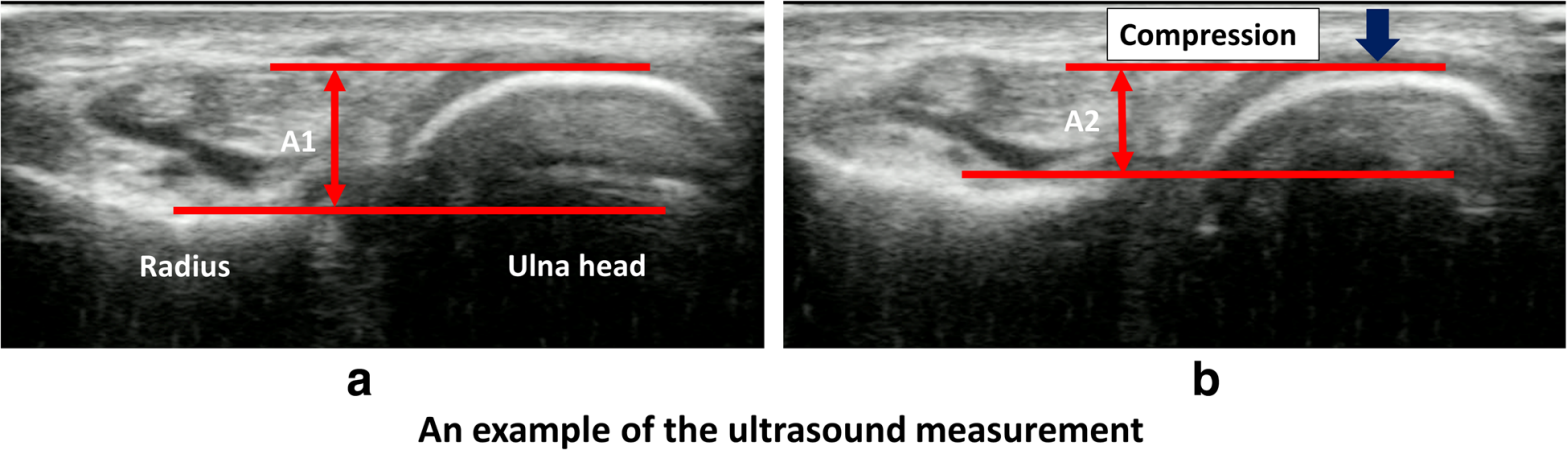
An example of an ultrasound measurement. **a** Initial position. A1: distance between the dorsal surfaces of the ulnar and the radius at the initial position. **b** Final position after compression. A2: distance between the dorsal surfaces of the ulnar and the radius at the final position after the compression. Arrow shows pressure to the ulna. A1–A2 were defined as the changes of radioulnar distance

**Fig. 3:**
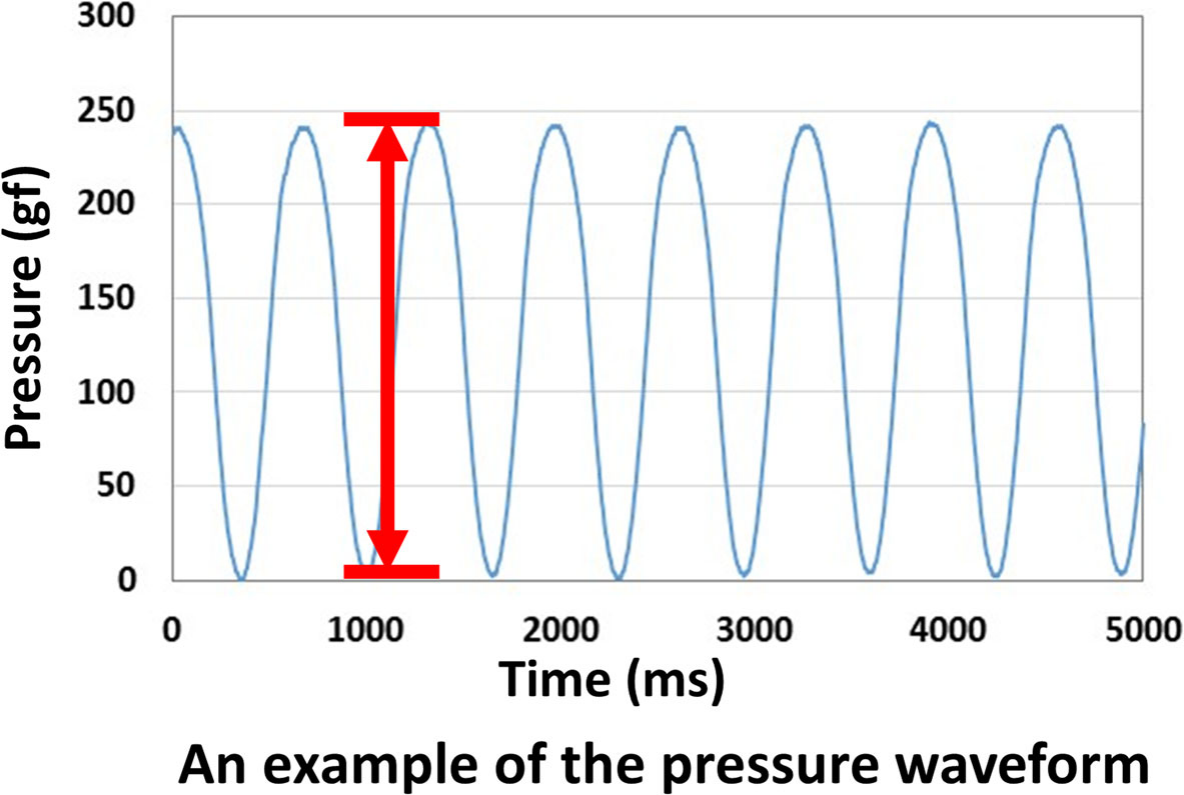
An example of the pressure waveform. The pressure at the initial placement of the transducer was taken as the baseline and the pressure when the transducer pressed down was measured

Using Analyze 10.0 Software (Biomedical Imaging Resource, Mayo Clinic, Rochester, MN), the recorded images were reviewed and the initial and final frames of the translation were chosen. The tangential line at the closest surface of the ulna to the transducer was defined. A parallel line to the ulna tangential line was drawn at the radius corner closest to the ulna. The distance from the transducer surface to the dorsal surface of the ulnar and radius was measured. In addition, the distance between the dorsal surface of the ulnar head and the dorsal surface of the distal radius was measured (Fig. 2). The first measurement was performed at the initial position (A1), and the second measurement was performed when the transducer pressed down the ulna so that the ulnar head shifted to the most palmar position (A2). At the same time, the pressure to the transducer was measured. The pressure at the initial placement of the transducer was taken as the baseline, and the pressure when the transducer pressed down was measured (Fig. 3). The changes in ulna distance, radius distance, radioulnar distance (=A1–A2, mm), pressure (gram force, gf), and pressure/distance ratio were measured for five times in each transducer displacement condition. The averages of five measurements were used for analysis.

### Statistical analysis

The results are expressed as mean ± standard deviation. The changes of radioulnar distance, pressure, and pressure/displacement ratio were compared among the different transducer displacements. One-way repeated measures ANOVA was used. All results were expressed as mean ± standard deviation. The parameters were considered statistically significant, when the *P* value was less than 0.05. All analyses were performed using Excel Statistics 2012 (SSRI Co., Tokyo, Japan) and SPSS Statistics (IBM, Tokyo, Japan) software.

## Results

**Fig. 4:**
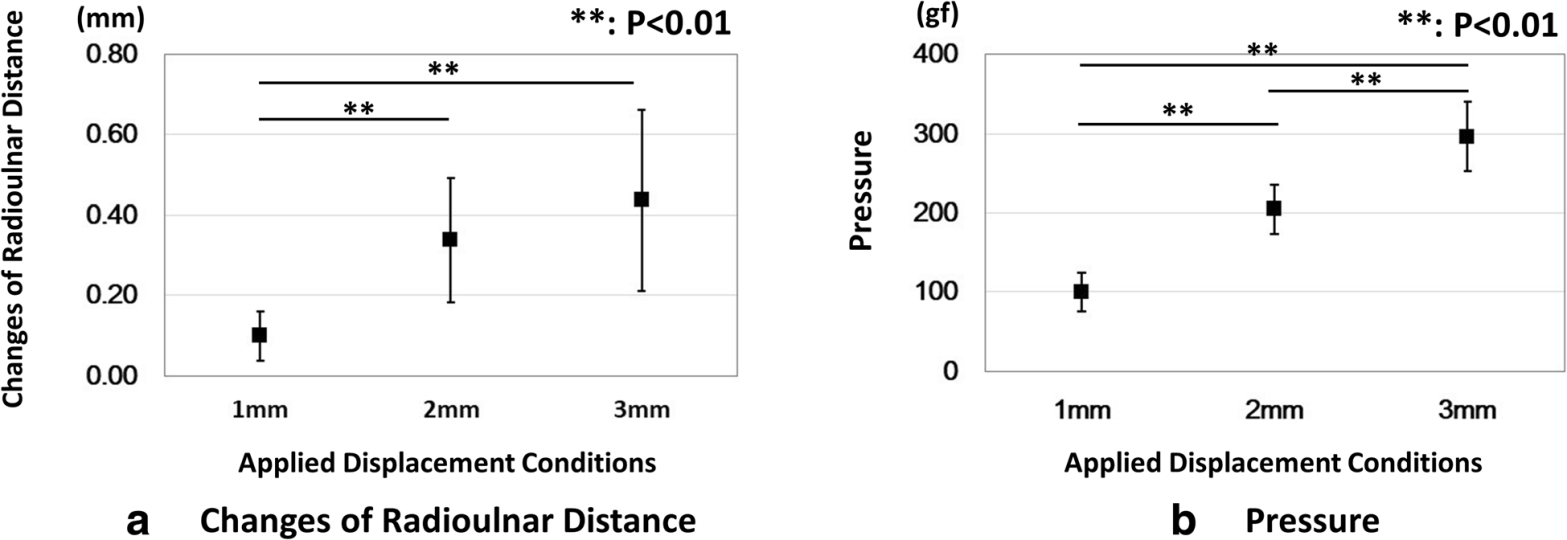
Results for the changes of radioulnar distance and the pressure. Horizontal axis indicates the displacement conditions of the transducer. **a** Changes of the radioulnar distance. **b** Pressure. **Showed significant differences between the conditions (*P* < 0.01)

The changes in radioulnar distances are shown in Fig. 4a. The changes of the ulna distances were 0.25 ± 0.29 mm, 0.27 ± 0.20 mm, and 0.38 ± 0.30 mm for the 1 mm, 2 mm, and 3 mm displacement conditions, respectively. The changes of the radius distances were 0.35 ± 0.29 mm, 0.60 ± 0.21 mm, and 0.81 ± 0.29 mm for the 1 mm, 2 mm, and 3 mm displacement conditions, respectively. The changes of the radioulnar distances were 0.10 ± 0.06 mm, 0.34 ± 0.16 mm, and 0.44 ± 0.22 mm for the 1 mm, 2 mm, and 3 mm displacement conditions, respectively. There were no significant differences between the conditions for the changes of ulna distances. The changes of radius distances were significantly increased as the transducer displacement became larger (*P* < 0.01). There was a smaller change in radioulnar distance in the 1 mm displacement condition compared to the other conditions (*P* < 0.05). There was no significant difference in the radioulnar distance between the 2 mm and 3 mm displacement conditions.

**Fig. 5:**
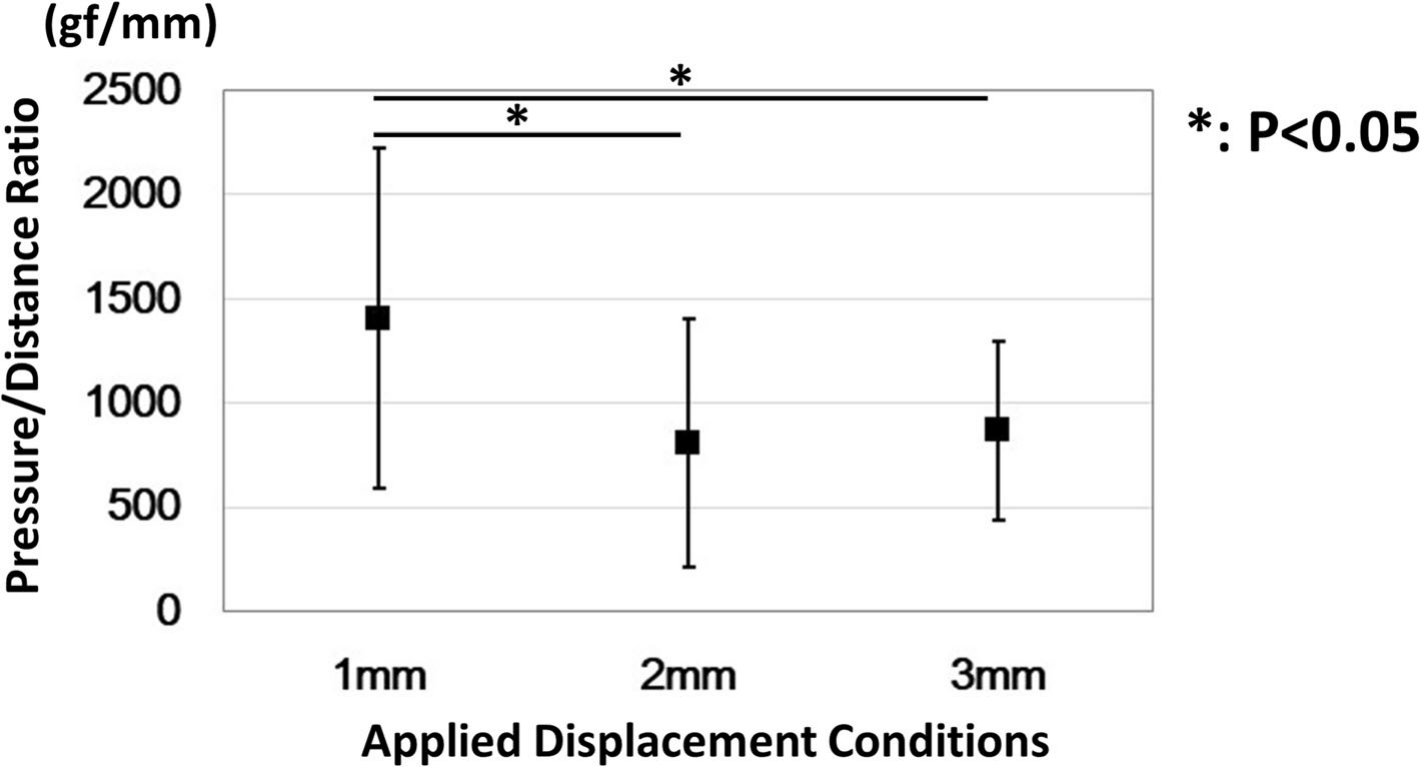
Results for the pressure/distance ratio. Horizontal axis indicates the displacement conditions of the transducer. *Showed significant differences between the conditions (*P* < 0.05)

The results of pressures are shown in Fig. 5. The pressures were 100.5 ± 24.4 gf, 205.2 ± 31.1 gf, and 296.8 ± 43.9 gf for the 1 mm, 2 mm, and 3 mm displacement conditions, respectively. The pressure/distance ratio results are shown in Fig. 5. The pressure/distance ratios were 1404.6 ± 815.8 gf/mm, 809.6 ± 597.0 gf/mm, and 867.0 ± 430.6 gf/mm for the 1 mm, 2 mm, and 3 mm displacement conditions, respectively. The pressure was significantly increased as the transducer displacement became larger (*P* < 0.01). There was a significant difference between the 1 mm displacement condition and the other 2 and 3 mm displacement conditions for the pressure/distance ratio (*P* < 0.05).

## Discussion

DRUJ instability has been recognized as one of the common clinical problems around the wrist. Several methods have evaluated the amount of dorsovolar translation based on CT [14, 22–26]. There were radioulnar ratio method [22], radioulnar line method [23], epicenter method [24], and subluxation ratio method [25]. However, these methods are difficult to reproduce, need a CT scan, and sometimes there were poor inter-observer agreements. It is also difficult to understand dynamic force-displacement conditions. In this study, we assessed the utility of a pressure-monitor ultrasound system as a noninvasive method to assess the degree of physiological dorsovolar translation in healthy volunteers. Diagnostic ultrasound equipment is widely available. The basic advantages of ultrasound, such as rapidity, noninvasiveness, portability, and low costs, are well known. In addition, using a pressure-monitor system, we could evaluate the DRUJ force-displacement relation.

Before enrolling patients with DRUJ instability, we wanted to study in detail the normal force-displacement relationship, and it was characterized in this study. Originally, it was expected that the amount of radioulnar translation would be dependent on the amount of force applied. However, the results of radioulnar distance showed that there were significant differences between the 1 mm displacement condition and the other 2 or 3 mm displacement conditions. There were no significant differences between the 2 mm and 3 mm displacement conditions. The pressure-distance ratio also showed the same tendency. This suggested that there was a smaller change in the radioulnar distance in the DRUJ under the 1 mm displacement condition. When displacements more than 2 mm were applied, the changes in the radioulnar distance became more obvious. At the same time, the ligaments and soft tissues around DRUJ started to stabilize the joint. Under displacement control, the capsuloligamentous structures of the joint were stretched and limited displacement through a restraining force. Therefore, there were no significant changes in the radioulnar distance between the 2 mm and 3 mm displacement conditions even when the applied pressure was increased. Thus, the application of 2 mm displacement and 200 g force were the critical stress for the capsuloligamentous structures to start stabilizing DRUJ.

Compared with previous cadaveric studies, the amount of translation was lower because of the good dynamic stability through muscular contraction [17–19]. Although it is difficult to compare the results directly, the ratio of displacement to the force was about three quarters compared to the previous study [19]. This may be because the examination was performed with remaining the mobility of the radius. As this clinical test intended to be applied to the patient, it was not performed in the condition that the subject felt uncomfortable. The advantage of using this system to evaluate DRUJ instability is to measure pressure and displacement simultaneously. In the case of the patients with DRUJ instability, the displacement may be induced with weaker force. Thus, even less than 3 mm displacement condition, it will be possible to evaluate the DRUJ instability with the force-displacement relationship. Because we evaluated the stability with a gradual increase in the application of stress, we could identify these subtle changes in vivo. This method may be useful to discriminate pathological conditions from normal conditions.

There are several limitations in our study. First, we evaluated DRUJ instability only in the forearm pronation position. There was a report which showed that there was no radioulnar instability in the forearm full pronation position if the radioulnar ligament was intact [21]. Patterns of instability are dependent on forearm position [6]. Other forearm positions may need to be evaluated in future studies. Second, ultrasound measurements are known to be operator- and experience-dependent. Even though we aimed to develop reliable equipment, an experienced operator is still needed for accurate image acquisition. Third, the small sample size may be another limitation. We limited the number of subjects so that we could investigate normal variance in detail before trying to investigate a larger number of subjects or patients. Now that we have confirmed the method and values from normal subjects, we can compare these to patients with DRUJ instability. In addition, we can compare these results with other examiners for a reliability test. Finally, the relationship between the stability and pathology of the DRUJ is still unknown. It would be helpful to understand the pathophysiology better by evaluating the relationship between DRUJ instability and physical findings.

## Conclusions

We used an ultrasound method to assess DRUJ stability by measuring changes in radioulnar distance and force application. This methodology and the indices of distance and pressure may be clinically useful to investigate the mechanical property of patients with DRUJ instability.

## Data Availability

The datasets used and/or analyzed during the current study are available from the corresponding author on reasonable request.

## Abbreviations

DRUJ: Distal radioulnar joint
TFCC: Triangular fibro cartilage complex
CT: Computed tomography
ANOVA: Analysis of variance
